# Meta-Analysis of the Changes of Peripheral Blood T Cell Subsets in Patients with Brucellosis

**DOI:** 10.1155/2018/8439813

**Published:** 2018-05-17

**Authors:** Rongjiong Zheng, Songsong Xie, Shaniya Niyazi, Xiaobo Lu, Lihua Sun, Yan Zhou, Yuexin Zhang, Kai Wang

**Affiliations:** ^1^Department of Infectious Diseases, The First Affiliated Hospital of Xinjiang Medical University, Urumqi, Xinjiang 830054, China; ^2^Department of Medical Engineering and Technology, Xinjiang Medical University, Urumqi, Xinjiang 830011, China

## Abstract

Brucellosis is one of the most prevalent zoonotic diseases in the world, but its pathogenesis is not very clear. At present, it is thought that it may be related to the immunity of T cells. The conclusions of related studies are inconsistent, and its clinical significance is not explicit. We searched published articles in electronic databases up to December 2017 identified as relating to the clinical features of human brucellosis in China. Only eight studies had sufficient quality for data extraction. Meta-analysis showed a significantly decreased proportion of CD4+ T cells in human brucellosis patients compared to healthy subject individuals. The frequency of CD8+ T cells was significantly higher in human brucellosis patients than that in the healthy control group. The pooled analysis presented a significant decrease of the CD4+/CD8+ ratio in human brucellosis patients compared to healthy subjects. There is immunologic dysfunction of T lymphocyte in patients with human brucellosis, the CD4+ and CD8+ T cells might be the important factors affecting the progress of brucellosis.

## 1. Introduction

Brucellosis is one of the most common zoonotic infections globally [1], which is a highly contagious zoonosis. Human brucellosis is transmitted to humans by direct/indirect contact with infected animals or through the consumption of raw meat and dairy products [2, 3]. Patients may have the symptoms of fever, sweating, fatigue, and osteoarthritis and even more serious conditions in different organ systems [4–6]. Brucellosis is often misdiagnosed as malaria, typhoid fever, rheumatic fever, osteoarthritis, and other diseases. Brucella has brought great harm to public health, food safety, and so on. The specific pathogenesis of brucellosis infection is not very clear. It is difficult to make a diagnosis by epidemiological and clinical symptoms. Human brucellosis is prone to multiple system complications, and once the brucellosis progresses to chronic phase, it will be difficult to cure [7]. Therefore, the pathogenesis of brucellosis is a hot research issue, and it is also believed that brucella infection is related to both innate immunity and adaptive immunity [8].

Studies have shown that the changes of T lymphocyte are crucial to the interpretation of the clinicopathological features of brucellosis in the process of chronic infection and recurrence [9, 10]. There are three mechanisms of acquired immunity in brucella infection: (1) the antimicrobial effect of macrophage induced by interferon-gamma secreted by CD4+, CD8+, *γ*, and *β* T lymphocyte [11]; (2) the hosts eliminate the macrophages infected by brucella through the cytotoxic effect of CD8+, *γ*, and *β* T lymphocytes [12]; and (3) the modulating effect of the antibody can enhance the phagocytosis of macrophages [13]. Activated CD8+ T lymphocytes and CD4+ Th1 type immune responses play an important role in the scavenging of intracellular pathogens. Treg has the function of inhibiting the proliferation of T lymphocyte and eliminating the pathogen. The changes in the number and function of Treg will inhibit the immune function of the host, which may be one of the mechanisms that leads brucella infection progresses into chronicity [14]. However, most of the immunology-related studies of the interaction between the host and brucella are mostly derived from domesticated ruminants or mice. The immune mechanism of brucella infection in diverse hosts is different.

There are few reports about the changes of peripheral blood T cell subsets in patients with brucellosis, and the results reported are not consistent [15–22]. It is difficult to draw a conclusion because of the deviations in experiments, population difference, and single or small sample study. In order to reduce the differences and bias among the various research institutes, meta-analysis method was used to analyze the previous research results to investigate the changes in the frequency of T cell subsets in peripheral blood of patients and provide the direction for further exploration of the mechanism of the immune pathogenesis of brucellosis.

## 2. Methods

### 2.1. Search Strategy

We performed a systematic review of the literature to identify articles relating to the changes of peripheral blood T cell subsets in patients with brucellosis. With the assistance of a professional medical librarian, we electronically searched for the literature in Wanfang Data, PubMed, and EMbase with MESH and keyword subject headings “brucellosis,” “Brucella,” “Brucel^∗^,” and “malta fever,” and “CD3,” “CD4,” “CD8,” “Th17,” “Th1,” “Th2,” “Treg,” and “Regulatory T Cells” for entries published from databases' inception before December 2017. We did not restrict the types of studies and publication languages. Duplicate entries were identified by two investigators screening the title and abstract of the article, the author, the year of publication, and the volume, issue, and page numbers of the source, and reviewed potentially all relevant articles.

### 2.2. Selection Criteria

We systematically and inclusively reviewed articles by two investigators. The reviewers selected articles firstly by title and abstract, next by full text, and lastly by analyzing eligible studies in detail until demonstrated 100% agreement in articles are included and excluded by two investigators.

Studies with the following criteria were excluded such as (i) articles related to nonhuman brucellosis, (ii) reported data that overlapped with already included articles, (iii) articles could not provide original data of the patients, (iv) and articles addressing topics not related to the changes of peripheral blood T cell subsets in patients with Brucellosis.

Studies with the following criteria were included such as (i) designed as a case-control study, (ii) the literatures assessed the changes in peripheral blood T cell subsets in patients with brucellosis, and (iii) provided sufficient data, including mean and standard deviation of T cell from case and control to calculate the efficient size.

### 2.3. Data Extraction

Data was extracted by two reviewers independently including date of the first author's name, publication year, study design, study location, patient, the number of male and female patients, detection technology, and treatment status methods of diagnosis, and results of each study were recorded. Study quality was assessed using the Newcastle-Ottawa scale (NOS). The results of data extraction must reach an agreement and consensus among the reviewers. Due to the inconsistency of the standard of the staging of disease in the literature included, the total mean and total standard deviation of the case group in the literature consist of acute and chronic staging are combined with the following formulas:

(1) $$\begin{array}{c} x=\frac{n_{1}x_{1}+n_{2}x_{2}+\cdots +n_{k}x_{k}}{n},\\ S=\sqrt{\frac{\left(n_{1}{x_{1}}^{2}+n_{2}{x_{2}}^{2}+\cdots +n_{k}{x_{k}}^{2}\right)+\left[\left(n_{1}-1\right){S_{1}}^{2}+\left(n_{2}-1\right){S_{2}}^{2}+\cdots +\left(n_{k}-1\right){S_{k}}^{2}\right]-{nx}^{2}}{n-1}}, \end{array}$$

where *n*_*i*_, *x*_*i*_, and *S*_*i*_ denote the number of samples, the mean, and the standard deviation of the *i*th group, respectively.

### 2.4. Statistical Analysis

Data were analyzed using the mean difference (MD) with 95% confidence intervals (CI) for continuous outcomes. The mean ± SD was extracted and calculated in all included publications. Cochran's Q test and Higgin's *I*^2^ statistics were simultaneously adopted for the test of heterogeneity of combined MDs. A random effects model was adopted to aggregate the pooled MD when significant heterogeneity existed (*p* < 0.1 and/or *I*^2^ > 50%); on the contrary, a fixed effects model was employed (*p* > 0.1 and/or *I*^2^ < 50%). Publication bias was detected by Egger's regression asymmetry test when the number of included trials ≥ 7. Sensitivity analyses were performed by omitting each study to identify the stability of combined results.

## 3. Results

### 3.1. Systematic Review

**Figure 1:**
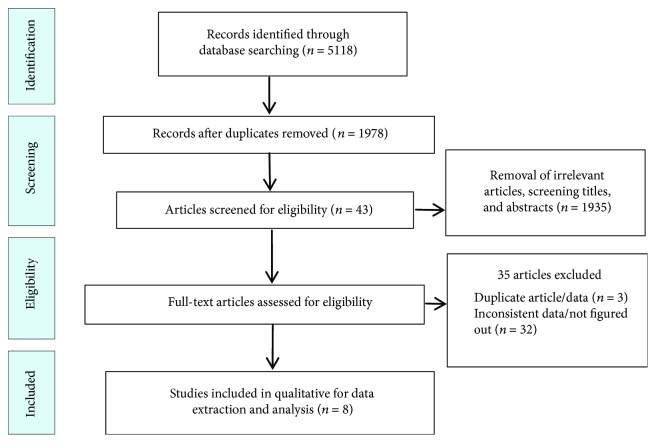
Procedure of the selection process.

Literature searches yielded 5118 potential articles, leaving 8 publications that met inclusion and exclusion criteria for data extraction and final analyses. Eight studies representing 396 patients with human brucellosis and 212 cases of healthy control were finally included in the meta-analysis. All 8 articles included in the analysis were case-control studies.  Figure 1 illustrates the detailed search process.

In the 8 studies we selected, there are 6 articles in English and 2 in Chinese. Seven studies provided data about CD3+ T cells in peripheral blood [15, 17–22], 8 studies are related to CD4+ T and CD8+ T cells in peripheral blood [17–22], 4 studies are related to the ratio of CD4+/CD8+ in peripheral blood [15, 16, 19, 20], and 3 studies are related to Th1 cells and Th2 cell [16, 17, 22]. Only 2 studies are related to Treg cells in peripheral blood. The basic characteristics of the literature and patients are shown in Table 1.

### 3.2. Changes of Peripheral Blood CD3+ T Cell

**Figure 2:**
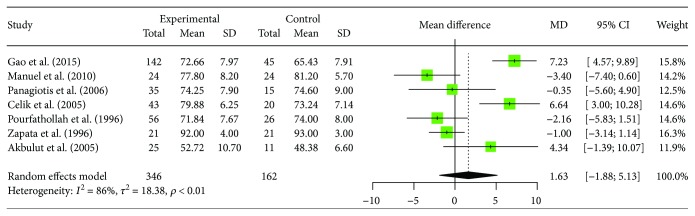
Forest plot of the changes of peripheral blood CD3+ T cell in human brucellosis patients compared with controls.

Seven studies including 346 patients with human brucellosis and 162 cases of healthy control, in which provided the data of the changes of peripheral blood CD3+ T cells in human brucellosis patients. Two studies reported that the proportions of CD3+ T cells in human brucellosis patients were significantly increased compared to control individuals [15, 19], while another 5 studies [17, 18, 20–22] showed that there was no significant difference between the two groups. The heterogeneity test showed (*I*^2^ = 85.6%, 95% CI (72.4%; 92.5%), *p* < 0.0001), indicating that there is statistical heterogeneity between studies. With regard to the heterogeneity results, random effects model was used. A meta-analysis showed that the proportions of CD3+ T cells in human brucellosis patients were increased but no significant difference between patients and control individuals ([MD = 1.6265, 95% CI (−1.8789; 5.1319), *p* = 0.3631]). The forest plot for these analyses was shown in Figure 2. Sensitivity analysis was included in the literature, and the effect of single study on the combined results was evaluated. The analysis showed that the results were not substantially altered, when any one study was deleted, as shown in Table 2. Egger's regression asymmetry test was used to evaluate publication bias, if any. No bias was found (*t* = 0.0210, df = 5, *p* = 0.9841).

### 3.3. Changes of Peripheral Blood CD4+ T Cell

Changes of peripheral blood CD4+ T cells were reported by 8 trials, containing 396 patients with human brucellosis and 212 cases of healthy control. In the 8 studies we selected, 6 studies reported that proportions of CD4+ T cells in human brucellosis patients were significantly decreased compared to control individuals [15–17, 19, 20, 22]; the other of 2 studies showed that there was no significant difference between the two groups [18, 21]. The result (*I*^2^ = 92.1%, 95% CI (86.8%, 95.3%), *p* < 0.0001) is showed by the heterogeneity test in the meta-analysis. Regarding this conclusion, the random effects model is applied.

**Figure 3:**
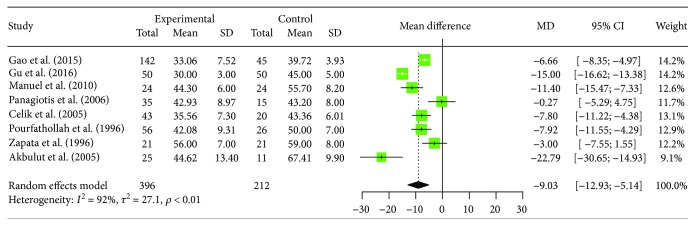
Forest plot of the changes of peripheral blood CD4+ T cell in human brucellosis patients compared with controls.

Results of the meta-analysis showed a significantly decreased proportion of CD4+ T cells in human brucellosis patients compared to healthy subject individuals ([MD = −9.03, 95% CI (−12.93; −5.14), *p* < 0.0001]). The forest plot for these analyses was shown in Figure 3. We also conducted sensitivity analysis to assess the influence of individual studies on the pooled results. The pooled results were not substantially altered, when any one study was deleted (Table 3). Egger's regression asymmetry test showed no evidence of publication bias (*t* = 0.5995, df = 6, *p* = 0.5708).

### 3.4. Changes of Peripheral Blood CD8+ T Cell

**Figure 4:**
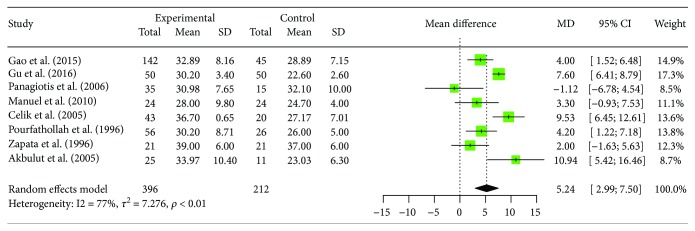
Forest plot of the changes of peripheral blood CD8+ T cell in human brucellosis patients compared with controls.

Eight trails with 396 patients with human brucellosis and 212 cases of healthy control reported changes of peripheral blood CD8+ T cell. These trials show homogeneity in the consistency of the trial results (*I*^2^ = 77.2%, 95% CI (54.7%, 88.5%), *p* < 0.0001). Therefore, a random effects model should have been used for statistical analysis. Meta-analysis showed that there is a significantly increased proportion of CD8+ T cell in human brucellosis patients compared to healthy subject individuals ([MD = 5.24, 95% CI (2.99; 7.50), *p* < 0.0001]). Five studies reported significantly increased proportions of CD8+ T cells in human brucellosis patients compared to control individuals [15, 16, 19, 20, 22]; the other 3 studies showed that there were no significant difference between the two groups [17, 18, 21]. The forest plots for these analyses were shown in Figure 4. Sensitivity analysis indicated that the above meta-analysis results were relatively stable, as shown in Table 4. Egger's regression asymmetry test showed no evidence of publication bias (*t* = −1.4357, df = 6, *p* = 0.2011).

### 3.5. Changes of Peripheral Blood CD4+/CD8+ Ratio

**Figure 5:**
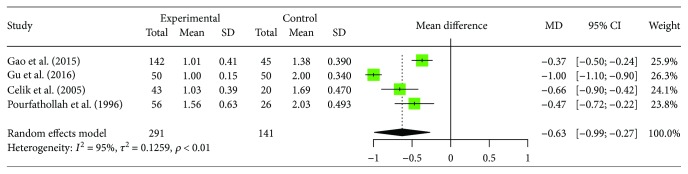
Forest plot of the changes of peripheral blood CD4+/CD8+ ratio in human brucellosis patients compared with controls.

Of the 8 included trials, 4 articles [15, 16, 19, 20] provided data of the changes of peripheral blood CD4+/CD8+ ratio in human brucellosis patients compared to controls including 291 patients with human brucellosis and 141 cases of healthy control. The trials showed homogeneity in the consistency of the trial results (*I*^2^ = 94.9%, 95% CI (90.0%, 97.4%), *p* < 0.0001). Therefore, a random effects model should have been used for statistical analysis. In the 4 studies we selected, each of them reported significantly decreased CD4+/CD8+ ratio in human brucellosis patients compared to control individuals. Results of the meta-analysis showed a significantly decreased proportion of CD4+/CD8+ ratio human brucellosis patients compared to healthy individuals ([MD = −0.6291, 95% CI (−0.99, −0.27), *p* = 0.0006]). The forest plot for these analyses was shown in Figure 5.

### 3.6. Changes of Peripheral Blood Th1 Cell

**Figure 6:**
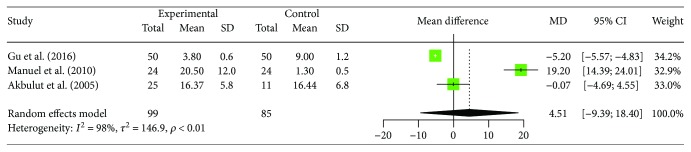
Forest plot of the changes of peripheral blood Th1 cell in human brucellosis patients compared with controls.

Changes of peripheral blood Th1 cell were also reported by 3 trials, which included 99 patients with human brucellosis and 85 cases of healthy control. In the 3 studies we selected, 1 reported significantly increased proportion of Th1 cells in human brucellosis patients compared to control individuals [16], 1 reported significantly decreased proportion of Th1 cell in human brucellosis patients compared to control individuals [17], and the rest of the 1 study found that there were no significant difference between the two groups [22]. The results of heterogeneity test (*I*^2^ = 98.1%, 95% CI (96.4%, 98.9%), *p* < 0.0001) indicate that there is statistical heterogeneity between studies. With regard to the heterogeneity results, random effects model was used. Results of the meta-analysis showed increased proportions of Th1 cells in human brucellosis patients but no significant difference between patients and control individuals ([MD = 4.51, 95% CI (−9.39, 18.40), *p* = 0.5249]). The forest plot for these analyses was shown in Figure 6.

### 3.7. Changes of Peripheral Blood Th2 Cell

**Figure 7:**
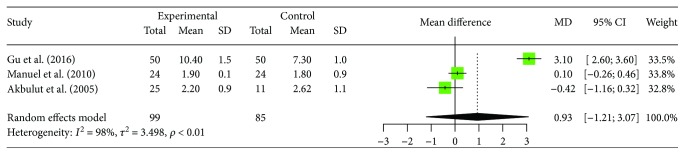
Forest plot of the changes of peripheral blood Th2 cell in human brucellosis patients compared with controls.

There are 3 articles providing data of the changes of peripheral Th2 cells in human brucellosis patients compared to controls including 99 patients with human brucellosis and 85 cases of healthy control. In the 3 studies we selected, 1 reported significantly increased proportion of Th2 cells in human brucellosis patients compared to control individuals [16]; the rest of the 2 studies found that there were no significant differences between the two groups [17, 22]. These trials show homogeneity in the consistency of the trial results [*I*^2^ = 98.1%, 95% CI (96.5%, 99.0%), *p* < 0.0001]. Therefore, a random effects model should have been used for statistical analysis. Results of the meta-analysis showed increased proportions of Th2 cell in human brucellosis patients but no significant difference between patients and control individuals ([MD = 0.93, 95% CI (−1.21, 3.07), p = 0.3925]). The forest plot for these analyses was shown in Figure 7.

## 4. Discussion

Although brucellosis is a disease that can be cured, there are still 5%~15% of brucellosis progression to chronicity with characteristics of a typical clinical manifestation, chronic fatigue syndrome, and recurrence [7, 23, 24]. Brucellosis is an infection-allergic zoonosis caused by brucella and has a worldwide distribution. There are more than 50 million new infections in the world every year. In recent years, the prevalence of brucellosis infection in China has also increased significantly [25]. Chinese CDC data showed that the annual incidence had increased from 5000 cases in 2002 to more than 60 thousand cases in 2015 [25]. In order to reveal the variation of T cell subsets such as CD3+ T cells, CD4+ T cells, CD8+ T cells, CD4+/CD8+ T cell ratios, Th1 cells, and Th2 cells in peripheral blood and further clarify its clinical significance, a total of 8 related articles were systematically evaluated in the study. All the researches were case-control studies. The quality of the research was evaluated by the NOS standard, and the overall score was 6~8. In general, the number of research in system analysis is not enough, the clinical studies of higher quality and large samples are needed to provide scientific and reliable evidence for the clinical application.

In recent years, the importance of T cells in brucella infection has been increasingly emphasized. Brucella was considered as an intracellular parasitic bacteria; in pathogenesis, cell-mediated immunity was the main reason [26, 27]. CD4+ and CD8+ T lymphocytes secrete cytokines such as interferon-gamma, TNF-alpha, and IL-2, which promote Th1 related to cytokine production, while the cytokine production and release by T lymphocytes can enhance the bactericidal ability of macrophages [28]. Interferon-gamma secreted by T lymphocytes not only activates the antibacterial ability of macrophages but also activates the cytotoxic effect of T lymphocyte [29]. Rafiei et al. found that the proportion of T lymphocytes secreting interferon-gamma in peripheral blood of chronic brucellosis patients was significantly lower than that in patients with acute stage, while the proportion of T lymphocytes producing IL-13 increased significantly [30]. Disproportions of Th1/Th2 and increases of cytokines secreted by Th2 cells in patients with chronic phase suggest that CD4+ T lymphocyte dysfunction is associated with chronic brucellosis. The proportions of CD4+ and CD8+ T lymphocytes in peripheral blood of patients with acute brucellosis were not significantly different from that of healthy controls, while the proportion of CD8+ T lymphocytes increased significantly in patients with chronic brucellosis, especially in patients with recurrent or symptomatic symptoms. But only a few CD8+ T lymphocytes secrete interferon [31]. Brucellosis mouse model inoculated with T lymphocytes could reduce the number of brucella in mice spleen, which indicates that CD4+ and CD8+ T lymphocyte immunity is involved in resistance to brucella infection [32]. MHC-II-deficient mice (without CD4+ T lymphocytes) had stronger ability to control S19 infection than MHC-I-deficient mice (without CD8+ T lymphocytes), indicating that CD8+ T lymphocytes play a major role in the immunity against brucellosis infection [33]. However, immunological studies on the interaction between host and brucella are mostly in cells or animal model experiments and cannot fully reflect the immune status of patients.

CD3+ T cells represent mature lymphocytes, which are the main active cells in cellular immunity, and CD3+ T cells represent the overall level of cellular immunity. By testing the changes of T lymphocyte subsets in 142 patients of brucellosis and 45 healthy controls, Gao et al. [15] has found that CD3+ T lymphocytes are increased in patients with brucellosis. Çelik et al. [19] reported that the proportion of CD3+ T lymphocytes in peripheral blood of patients with acute brucellosis was significantly increased. Some other researches showed that the numbers of peripheral blood CD3+ T lymphocytes had no significant difference between brucellosis patients and healthy controls. This study showed that CD3+ T cells in human brucellosis patients had increased but there were no significant difference between patients and control individuals. The conclusion that we obtained needs to be confirmed further.

CD4+ T cell is an auxiliary T lymphocyte that assists other cells to participate in the immune response. The decrease of CD4+ T cells can lead to a series of dysfunction in Tc, NK, macrophages, and B cells. CD8+ T cells are the main effector cells that play specific cytotoxic effects, specifically killing target cells, mainly involved in the resistance to intracellular infection [34] and antitumor [35] and participate in graft rejection [36]. CD8+ T cells are not just a homogeneous cell group, some part of them are immunosuppressive cells that inhibit the function of other immune cells [37]. The ratio of CD4+/CD8+ is related to the function of the effector T cells, usually keeping dynamic balance in order to maintain the stability of cellular immune function. The decrease in the number of CD4+ T cells and the ratio of CD4+/CD8+ can lead to the dysfunction of the effector T cells. The conclusions from different related researches of the changes in the percentage of CD4+ T, CD8+ T, and CD4+ T/CD8+ T ratios in peripheral blood in patients with brucellosis are not consistent. According to the results of the rat model, it is conjectured that the CD8+ T lymphocyte actives as CTL and plays the cytotoxic effect by secreting granulysin [38]. Some scholars [39] also believed that it may only compensate for the ineffective CD4+ T lymphocyte response by increasing the number of CD8+ T lymphocyte in patients with chronic brucellosis. Some studies reported that the CD4+ T lymphocyte proliferation reaction ability in patients with chronic brucellosis was significantly lower than that of the healthy controls and the acute patients [40]. This study shows that the frequency of CD4+ T cells in brucellosis patients is lower than that of healthy controls, while the CD8 cell frequency is higher than that of controls, and the ratio of CD4/CD8 is lower than the control group, which shows that there is immunologic dysfunction of the T lymphocyte in patients with brucellosis. Further research is needed to confirm the changes of CD4+ and CD8+ T cell function in peripheral blood of patients with brucellosis.

Th1 and Th2 cells are the first classified CD4+ T cell functional subgroups. Th1 cells mainly mediate cellular immunity and delayed hypersensitivity through secreted inflammatory cytokines. Th2 cells mainly mediate humoral immune response, and both of them must keep dynamic balance in order to maintain the stability of cellular immune function. The IFN-gamma secreted by Th1 cells antagonized the IL-4 secreted by Th2 cells, and IFN- gamma secreted by Th1 cells will destroy the macrophages infected with brucella [41–44]. Dorneles et al. [45] indicated that prime-immunized with *B. abortus* S19 or RB51 in cattle induce a strong and complex Th1 immune response characterized by proliferation of CD4+ Th1 cells and higher secretion of IFN-*γ*, which plays an important role in controlling the occurrence and development of brucella infection. Studies have shown that immune response induced by Th1 cells is necessary for highly effective vaccines to prevent brucellosis. Therefore, the increase of Th2 cell level in host cells may inhibit the immune response of Th1 cells and break the balance between Th1 and Th2 cells, leading the occurrence of brucellosis [46]. This study shows that proportions of Th1 and Th2 cells increased in human brucellosis patients but neither of them has significant difference between patients and control individuals. The statistical power of the analysis was not enough since only 3 studies were used for this part of the analysis; the conclusion that we obtained still needs further research.

Treg cells play an important role in preventing immune responses against pathogens [47]. Abbas et al. found that the numbers of CD4+ Treg cells and their CD25^high^ and FoxP3^high^ subsets increase significantly in the peripheral blood of human brucellosis, with this increase being greater in the chronic group [48]. By contrast, Ganji at al. found a significantly lower percentage of CD25/FoxP3+ Treg cells in chronic patients than in the acute patients and control groups [49]. Only 2 eligible studies are related to Treg cells in peripheral blood, which is unable to carry out the meta-analysis.

Th17 cells have been found to be major stimulatory participants in the pathogenesis of human disease [50]. In mouse models, Pujol et al. found that the activation of Th17-related response was ineffective to control the *B. canis* infection [51]. There were no reports on Th17 in patients with brucellosis.

Our study has some limitations. First, heterogeneities exist among the included documents, which may be related to the diversities of race, age, diagnostic criteria and the staging of acute and chronic illness. Second, it is hard to unify the standard staging of acute and chronic illness with the included literatures; the results of the case group in the literature consist of acute and chronic staging are combined with the formulas, leading to the failure to do more accurate analysis for the different stages of the brucellosis patients. Last, due to the quantity of the included literature is not enough, the subgroup analyses cannot be tested.

In summary, we found that there was immunologic dysfunction of T lymphocyte in patients with human brucellosis, which may provide some pieces of evidence for immune regulation therapy of brucellosis. More high-quality and large sample experiments are needed to further confirm the relationship between the frequency and function of peripheral blood T cells subsets and the pathogenesis of brucellosis.

## Figures and Tables

**Table 1:** Characteristics of studies included in the meta-analysis.

Year	Author(s)	Region	Treatment status	Study design	Number		Sex (M/F)		NOS scale
					Patient	Control	Patient	Control	
2015	Gao et al. [15]	China	No report	Case-control design	142	45	89/53	27/18	7
2016	Gu et al. [16]	China	No report	Case-control design	50	50	42/8	38/12	7
2010	Manuel et al. [17]	Spain	All patients were treated	Case-control and cohort design	24	24	17/7	18/6	8
2006	Panagiotis et al. [18]	Greece	No report	Case-control design	35	15	28/7	11/4	7
2005	Celik et al. [19]	Turkey	All patients were treated	Case-control and cohort design	43	20	19/24	11/9	8
1996	Pourfathollah et al. [20]	Iran	No report	Case-control design	56	26	—	15/11	6
1996	Zapata et al. [21]	Spain	All patients were treated	Case-control and cohort design	21	21	19/2	—	6
2005	Akbulut et al. [22]	Turkey	No report	Case-control design	25	11	16/9	7/4	7

**Table 2:** The results of CD3+ T cell data sensitivity analysis.

Influential analysis (random effects model)					
	MD	95% CI	*p* value	Tau^2^	*I* ^2^
Omitting 1	0.5238	[−2.5631; 3.6106]	0.7395	10.5972	74.9%
Omitting 2	2.4580	[−1.2836; 6.1997]	0.1979	17.8897	85.7%
Omitting 3	1.9108	[−1.9972; 5.8188]	0.3379	20.2834	87.8%
Omitting 4	0.7666	[−2.9579; 4.4912]	0.6866	17.5758	85.1%
Omitting 5	2.2725	[−1.6304; 6.1755]	0.2538	19.6843	86.5%
Omitting 6	2.1258	[−1.9855; 6.2371]	0.3109	21.8144	84.7%
Omitting 7	1.2547	[−2.6029; 5.1123]	0.5238	19.8369	87.8%

**Table 3:** The results of CD4+ T cell data sensitivity analysis.

Influential analysis (random effects model)					
	MD	95% CI	*p* value	Tau^2^	*I* ^2^
Omitting 1	−9.4574	[−14.0627; −4.8521]	<0.0001	33.4726	91.0%
Omitting 2	−7.8215	[−11.0090; −4.6339]	<0.0001	13.7059	80.3%
Omitting 3	−8.7126	[−13.0720; −4.3533]	<0.0001	30.0784	93.2%
Omitting 4	−10.1635	[−14.0935; −6.2334]	<0.0001	23.9963	91.9%
Omitting 5	−9.2435	[−13.6663; −4.8207]	<0.0001	30.8919	93.1%
Omitting 6	−9.2212	[−13.6181; −4.8242]	<0.0001	30.5423	93.1%
Omitting 7	−9.8610	[−13.9606; −5.7614]	<0.0001	26.2844	92.4%
Omitting 8	−7.6681	[−11.5829; −3.7532]	0.0001	24.6279	92.3%

**Table 4:** The results of CD8+ T cell data sensitivity analysis.

Influential analysis (random effects model)					
	MD	95% CI	*p* value	Tau^2^	*I* ^2^
Omitting 1	6.7050	[5.7618; 7.6482]	<0.0001	8.3543	77.5%
Omitting 2	4.8388	[3.5210; 6.1566]	<0.0001	8.5000	71.9%
Omitting 3	6.5501	[5.6575; 7.4427]	<0.0001	5.7473	74.8%
Omitting 4	6.5028	[5.6013; 7.4042]	<0.0001	7.5933	79.0%
Omitting 5	6.0810	[5.1607; 7.0012]	<0.0001	7.4068	77.1%
Omitting 6	6.5711	[5.6481; 7.4941]	<0.0001	8.3139	78.9%
Omitting 7	6.6377	[5.7287; 7.5466]	<0.0001	6.5298	75.8%
Omitting 8	6.2442	[5.3510; 7.1373]	<0.0001	7.1158	78.5%
